# Glycosylation of *Candida albicans* Cell Wall Proteins Is Critical for Induction of Innate Immune Responses and Apoptosis of Epithelial Cells

**DOI:** 10.1371/journal.pone.0050518

**Published:** 2012-11-30

**Authors:** Jeanette Wagener, Günther Weindl, Piet W. J. de Groot, Albert D. de Boer, Susanne Kaesler, Selvam Thavaraj, Oliver Bader, Daniela Mailänder-Sanchez, Claudia Borelli, Michael Weig, Tilo Biedermann, Julian R. Naglik, Hans Christian Korting, Martin Schaller

**Affiliations:** 1 Department of Dermatology, Eberhard Karls University Tübingen, Tübingen, Germany; 2 Department of Pharmacology and Toxicology, Institute of Pharmacy, Freie Universität Berlin, Berlin, Germany; 3 Regional Center for Biomedical Research, Albacete Science & Technology Park, University of Castilla – La Mancha, Albacete, Spain; 4 Department of Medical Microbiology and National Reference Center for Systemic Mycoses, University Medical Center Göttingen, Göttingen, Germany; 5 Department of Oral Immunology, King’s College London Dental Institute, King’s College London, London, United Kingdom; 6 Department of Dermatology, Ludwig-Maximilians-University, München, Germany; 7 Aberdeen Fungal Group, Institute of Medical Sciences, University of Aberdeen, Aberdeen, United Kingdom; National Jewish Health and University of Colorado School of Medicine, United States of America

## Abstract

*C. albicans* is one of the most common fungal pathogen of humans, causing local and superficial mucosal infections in immunocompromised individuals. Given that the key structure mediating host-*C. albicans* interactions is the fungal cell wall, we aimed to identify features of the cell wall inducing epithelial responses and be associated with fungal pathogenesis. We demonstrate here the importance of cell wall protein glycosylation in epithelial immune activation with a predominant role for the highly branched *N*-glycosylation residues. Moreover, these glycan moieties induce growth arrest and apoptosis of epithelial cells. Using an *in vitro* model of oral candidosis we demonstrate, that apoptosis induction by *C. albicans* wild-type occurs in early stage of infection and strongly depends on intact cell wall protein glycosylation. These novel findings demonstrate that glycosylation of the *C. albicans* cell wall proteins appears essential for modulation of epithelial immunity and apoptosis induction, both of which may promote fungal pathogenesis *in vivo*.

## Introduction

Innate immunity plays the vital role of ‘gatekeeper’ of mucosal epithelia and is required to maintain homeostatic function and to coordinate immunological reactions against commensal and pathogenic microbes [1], [2]. The specialized and complex interaction between microbes, epithelial cells and local immune cells results in either a degree of mutualism (commensalism) or a breach of the mucosal barrier and subsequent cell injury (pathogenicity). Of particular interest are ‘opportunistic’ microbes such as *Candida albicans*, the most common fungal pathogen of humans, which normally colonizes mucosal surfaces as a harmless saprophyte but can cause mucosal disease in a significant proportion of immunocompromised individuals.

Contact with epithelial cells is mediated by the fungal cell wall, which is an essential structure that provides physical strength and protects the fungus from hostile environments (reviewed in [3], [4]). The cell wall consists of an inner layer of chitin and β-glucan and an outer layer of densely packed mannoproteins. Both β-glucans and mannoproteins derived from the *C. albicans* cell wall are known to stimulate myeloid cells through the activation of toll-like receptors (TLRs), particularly TLR2 and TLR4, C-type lectin receptors such as dectin-1, and mannose receptor (MR) (reviewed in [5]). Cytokine secretion in myeloid cells was shown to be mediated by three specific *C. albicans* cell wall components: *N*-linked mannans via MR, *O*-linked mannans via TLR4, and β-glucans via dectin-1/TLR2 [6]. It has been proposed that during murine systemic infections immune activation occurs due to the gradual exposure of the underlying β-glucan after removal of surface mannoproteins from yeast and hyphal cells. In support of this hypothesis, caspofungin treatment of *C. albicans* (an antifungal drug that targets the cell wall), mediated unmasking of β-glucan moieties resulting in enhanced immune reactivity via dectin-1 stimulation [7]. This model suggests that during systemic infections the surface mannoproteins may “shield” the fungus from immune attack by preventing β-glucan recognition.

Although integrated models for how *C. albicans* is recognized and targeted by myeloid cells are available [8], a great deal less is known about how epithelial cells and mucosal tissues interact with the fungus. Using an in vitro model of oral candidosis based on reconstituted human epithelium (RHE), we previously demonstrated that infection with *C. albicans* induces the expression of IL-8, GM-CSF, IL-1alpha, IL-1beta, IL-6, IFN-gamma and TNFalpha [9]. Subsequently, in the same model, we showed that activation of this pro-inflammatory response by *C. albicans* results in recruitment of polymorphonuclear (PMN) cells and protection against fungal invasion and infection in a toll-like receptor (TLR)4-dependent manner [2]. Others have also confirmed that *C. albicans* stimulates cytokine production in mucosal monolayer cell lines and primary mucosal cells [10], [11], [12], [13], [14], [15], [16]. However, despite recent progress, the nature of *C. albicans* surface moieties responsible for epithelial cell immune activation is undefined.

Here, we report that glycan moieties of *C. albicans* cell wall proteins are critical for epithelial-fungal interactions and the induction of innate immune responses. Furthermore, we propose that these glycan moieties promote fungal pathogenesis by inducing cell cycle arrest and apoptosis in mucosal epithelial cells.

## Materials and Methods

### Strains, Media and Growth Conditions

*C. albicans* wild-type strain SC5314, *mnt1Δ/mnt2Δ, pmr1Δ and och1Δ* glycosylation mutants and *S. cerevisiae* wild-type strain were used (overview of strains with phenotype and references given in Table 1) and maintained on Sabouraud’s dextrose agar (Difco).

**Table 1:** Fungal strains used in this study.

strain	phenotype	reference
*C. albicans* SC5314	Wild-type strain	[50]
*C. albicans och1Δ*	defective in *N*-mannosylation due to loss of Och1 (α-1,6-mannosyltransferase),attaching first mannose residues to *N*-mannan structure	[25]
*C. albicans mnt1Δ mnt2Δ*	defective in *O*-mannosylation due to loss of Mnt1 and Mnt2 attaching secondand third α-1,2-mannans to proteins	[27]
*C. albicans pmr1Δ*	defective in *N*- and *O*-mannosylation due to loss of Pmr1, essential cofactor forMnt and Och1 enzymes	[26]
*S. cerevisiae* BY4741	Wild-type strain	[51]

### Cell Wall Preparations

*C. albicans* cell walls were isolated as described elsewhere [17]. The “SDS/β-Me” fraction represents material that is extracted by incubating broken cell fragments, after repeated washing with 1 M NaCl, with SDS/β-mercaptoethanol extraction buffer (2% SDS, 150 mM NaCl, 100 mM Na-EDTA, 100 mM β-mercaptoethanol, and 50 mM Tris-HCl, pH 7.8) for 5 min at 100°C followed by centrifugation. After a second SDS/β-mercaptoethanol extraction and repeated washing with milliQ water, the SDS/β-mercaptoethanol-treated water-insoluble cell walls were freeze-dried. For obtaining more defined cell wall (protein) fractions (CWFs) the isolated walls were incubated with either HF-pyridine, endo-β-1,3-glucanase and/or endo-β-1,6-glucanase as described [17]. After each incubation the solubilized fractions were dialyzed overnight against milliQ water and freeze-dried. Prior to use, cell walls and CWFs were normalized to the amount of *Candida* cells used for cell wall isolation.

Protein degradation was performed by proteinase K (New England BioLabs) digestion (cell wall/proteinase K ratio 50∶1, w/w) for 30 min at 37°C. For protein deglycosylation, cell walls were incubated with 25 U PNGaseF (New England BioLabs) per 1 µg cell wall for 1 h at 37°C or with 1 volume 0.1 M NaOH for 6 h at room temperature through orbital shaking.

### Ethic Statement

C57BL/6 wild-type mice were purchased from Charles River (Sulzfield, Germany), TLR2-deficient mice were a kind gift from C. Kirschning (Technical University Munich), TLR4-deficient and MyD88-deficient mice were kindly provided by Dr. S. Akira (Osaka University). TLR2/4-deficient mice were generated by mating TLR2-deficient mice with TLR4-deficient mice. All deficient strains were in the C57BL/6 background. The animals were bred under specific pathogen-free conditions at the animal facility of the University of Tübingen according to European guidelines (FELASA) and to the guidelines for the care and use of laboratory animals of the German Animal Protection Law. Protocols were approved by the board institution animal facility of the University of Tübingen and the local authorities Regierungspräsidium Tübingen with the permit numbers §4 Abs. 3 Az v. 25.04.07 and Az. v. 05.06.09 according to German Animal Protection Law.

### Cell Culture and Stimulation

For infection studies and experiments with *C. albicans,* cell walls and CWFs the human buccal carcinoma cell line TR146 was used [18]. Cells were cultured in D-MEM medium with 10% FCS and 0.1% gentamicin solution (50 mg/ml) at 37°C in 5% CO_2_. Infection studies were performed in antibiotic and antimycotic free culture medium. For receptor inhibition human epithelial cells (TR146) were pre-incubated with 10 µg/ml functional-grade neutralizing anti-human TLR2 mAb TLR2.1, anti-human TLR4 HTA125, anti-human CD206 (mannose receptor c type 1) (AbD Serotec), IgG2a (eBiosciences) and IgG1 isotype control (AbD Serotec), 100 µg/ml laminarin or 40 µg/ml *S. c.* mannan (Sigma-Aldrich) 2 h prior to stimulation with *C. albicans* cell walls and for endocytosis blocking human epithelial cells (TR146) were pre-incubated with 5 µM cytochalasin D (Sigma) for 30 min. Primary cultures of murine epithelial cells were obtained from oral mucosa. After overnight treatment of oral biopsies upside-down in trypsin solution at 4°C, the epidermis was separated from the dermis and the epidermal cells collected by centrifugation. Murine epithelial cells were initially cultured in defined medium (64.5% D-MEM, 21.5% Ham’s, 10% fetal calf serum, 2% penicillin-streptomycin (10000 U/ml, 10 mg/ml)) for 2 days at 37°C in 5% CO_2_, followed by culture in a second defined medium (94% MCDB, 2% fetal calf serum, 2% penicillin-streptomycin) for 3 days at 37°C in 5% CO_2_ prior to experimentation.

### RHE Infection Model

For three-dimensional skin models, 1×10^6^ human oral epithelial cells (TR146) were seeded on inert filter substrates (Nunc, polycarbonate filter, 0.4 mm pore size, 0.5 cm^2^) in antibiotic/antimycotic-free defined keratinocyte growth medium (KGMgold, Lonza) for 9 days. After 5 days inert filter substrates were lifted to the air–liquid interface and basal cells were fed through the filter substratum. Epithelium was infected on day 9 with 2× 10^6^ *C. albicans* yeasts for 4 h to 24 h.

### RNA Isolation and Quantitative RT-PCR

Total RNA was extracted using the NucleoSpin RNAII kit (Macherey-Nagel) and cDNA was synthesized using 1 µg total RNA with SuperScript III Reverse Transcriptase (Invitrogen). Amplification of cDNA was performed as described previously [2]. Cytokine and TLR gene expression was normalized to the housekeeping gene and analyzed using the comparative Ct method (ΔΔCt). The fold change in the target genes were calculated using the equation 2^−ΔΔCt^ and data is presented as fold mRNA increase. Reactions were performed in duplicate.

### Immunoblot

Epithelial cells were lysed using whole cell lysate buffer (1 mM EDTA, 0.005% (v/v) Tween-20, 0.5% (v/v) Triton X-100, 5 mM NaF, 6M Urea) freshly supplemented with protease and phosphatase inhibitors (25 µg/ml leupeptin and pepstatin, 100 µm PMSF, 3 µg/ml aprotinin, 2 mM sodium-*ortho*-vanadat), left on ice for 30 min, and centrifuged for 5 min in a refrigerated microfuge. Supernatants were assayed for total protein using the Roti®Quant universal kit (Carl Roth, Karlsruhe) according to the manufacturers’ instructions.

Protein extracts (10 µg total protein per sample) were separated by SDS-PAGE according to the method of Laemmli [19] on a Biorad Protean II system, before transferred to PVDF membranes (Hybond™-P, GE Healthcare) for 60 min using a semi-dry transfer system. After probing with primary antibodies (anti-TLR4 (clone H-80, Santa Cruz) or anti-β-actin (clone 13E5, Cell Signaling)) and secondary antibodies, membranes were developed using LumiGlo™ chemiluminescent substrate (Cell Signaling) and exposed to ECL film (GE Healthcare).

### Cytokine Analysis

Human GM-CSF, IL-6 and IL-8 and mouse MIP-2 concentrations in the culture supernatants were determined using commercially available ELISA Kits (DuoSet, R&D Systems).

### Light Microscopy

For each oral RHE, 5 µm sections were prepared using a Leica RM2055 microtome and silane-coated slides. After dewaxing in xylene, endogenous peroxidise activity was blocked using 3% (v/v) hydrogen peroxide (H_2_O_2_). Antigen retrieval was undertaken by microwaving the sections at 960 W for 20 min in citrate buffer (100 mmol/L, pH 6.0 NaoH). Cleaved (activated) caspase-3 expression was determined using a rabbit polyclonal anti-active caspase-3 antibody (R&D Systems, 1∶100) and counterstained with peroxidase conjugated goat anti-rabbit secondary IgG antibody, followed by diaminobenzidine (DAB) chromogen detection as per manufacturer’s protocol. To visualize *C. albicans*, sections were stained using Periodic Acid Schiff (PAS), counterstained with haematoxylin and examined by light microscopy.

### Confocal Microscopy

Cells seeded on culture slides (BD Falcon) were incubated for 24 or 48 h with *C. albicans* cell walls, fixed with periodate lysine paraformaldehyde PLP and permeabilized with 0.5% Triton X-100. Non-specific binding was blocked using 5% donkey serum. Samples were stained using anti-p27^kip1^ (Santa Cruz Biotechnology) or anti-Caspase-3 (active form, R&D Systems) followed by detection with the fluorochrome-coupled donkey anti-rabbit-Cy3 secondary antibody (Dianova). Nuclei were stained with TOPRO (Invitrogen). For confocal microscopy of oral RHE specimens, tissue was cryofixed in liquid nitrogen, and 5-µm sections were placed on silan-coated slides. Sections were fixed in PLP (paraformaldehyde and lysine in PBS) for 2 min, followed by incubation with PBS for 5 min, PBS/BSA (0.1%) plus Tween 20 (0.1%) for 10 min, and PBS plus 10% donkey serum for 30 min at room temperature. Anti- (activated) caspase-3 polyclonal rabbit antibody (R&D Systems, 1∶100) and human anti–*C. albicans* serum (1∶60; Virion\Serion) were added for 60 min at room temperature. Sections were then incubated with donkey anti-rabbit–Dy549 (1∶800; Dianova) and donkey anti-human–Cy5 (1∶500; Dianova) for 60 min. All nuclei were stained with TOPRO (Invitrogen). All washing and antibody addition steps were performed with a combination of PBS, BSA, and Tween. The sections were analyzed with a confocal laser scanning microscope (Leica TCS SP; Leica Microsystems) at × 40 magnifications. To determine the relative levels of caspase-3 protein expression, fluorescence intensity measurements were performed on the confocal images using the Leica PowerScan software.

### Proliferation and Apoptosis Assays

Proliferation of human epithelial cells was measured using [^3^H]thymidine (GE Healthscare) for DNA labeling. Cultured cells were pre-treated 6 h with *C. albicans* cell walls before incubation with [^3^H]thymidine (1 µCi/ml) for a further 18 h. Incorporation was measured using a beta-counter (PerkinElmer). Proliferation of mouse oral epithelial cells was analyzed using a colorimetric Cell Proliferation ELISA (BrDU, Roche). For apoptosis analysis, Annexin V-FITC Apoptosis Detection Kit (BD Pharmingen) was used.

### Endotoxin, SDS and β-mercaptoethanol Quantification

Endotoxin contamination in cell wall and CWF preparations was analyzed using the limulus amebocyte lysate (LAL) assay (QCL-1000; Lonza) and was less than 1 EU/ml (<100 pg/ml). SDS contamination was analyzed as previously described [20] and was lower than 0.001% SDS (w/v). β-mercaptoethanol was quantified using Ellman’s Reagent [21] and could not be detected in the cell wall and CWF preparations.

### Statistics

All experiments were performed at least 3 times and revealed comparable results. Results are presented as mean ± SEM of three biological replicates. Statistical significance was determined using the 2-tailed paired Student’s *t* test. A *P* value of 0.05 or less was considered significant.

## Results

### *C. albicans* Cell Walls Induce Epithelial Cytokines and TLR4 Expression

**Figure 1:**
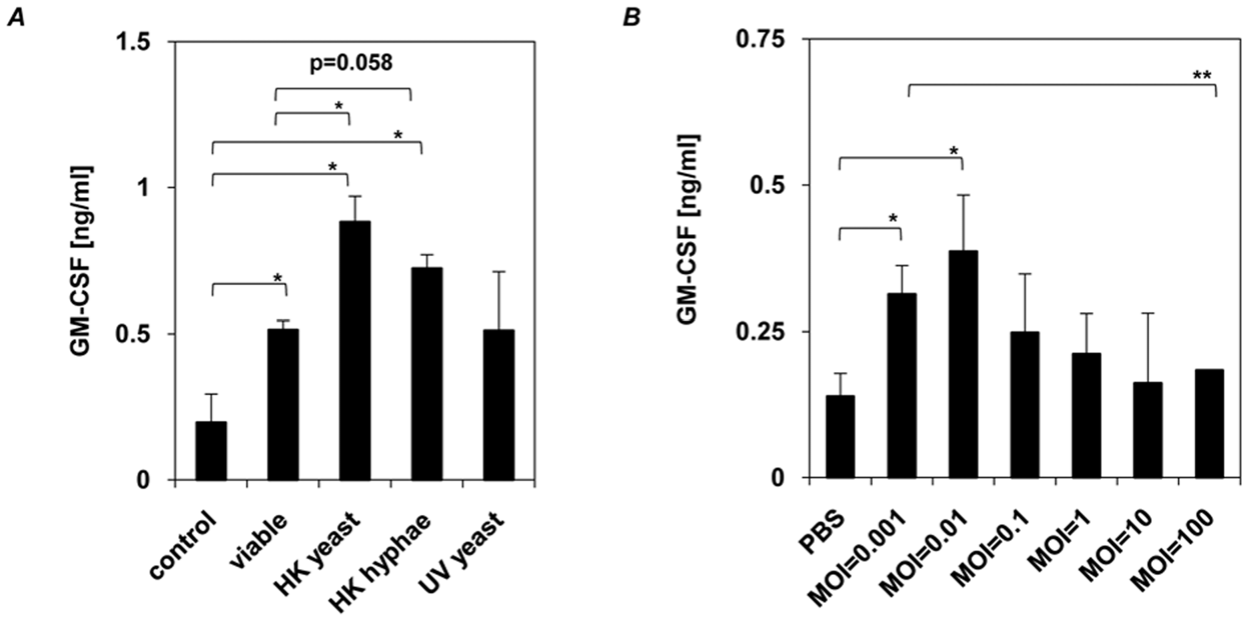
Effector responses of human oral epithelial cells to *C. albicans*. *C. albicans* induced GM-CSF secretion is independent of morphology, but dependent on fungal burden. (**A**) Human epithelial cells (1×10^6^ cells) were incubated with viable *C. albicans*, heat−/UV-killed yeast and heat-killed hyphae (1×10^6^ cells) for 24 h and (**B**) increasing numbers of viable *C. albicans* (1×10^3^ to 1×10^8^ cells, MOI = 0.001 to 100) for 24 h. (**A** and **B)**, *n* = 3 (± *SEM*), * p<0.05, ** p, 0.01, 2-tailed paired Student’s *t* test.

**Figure 2:**
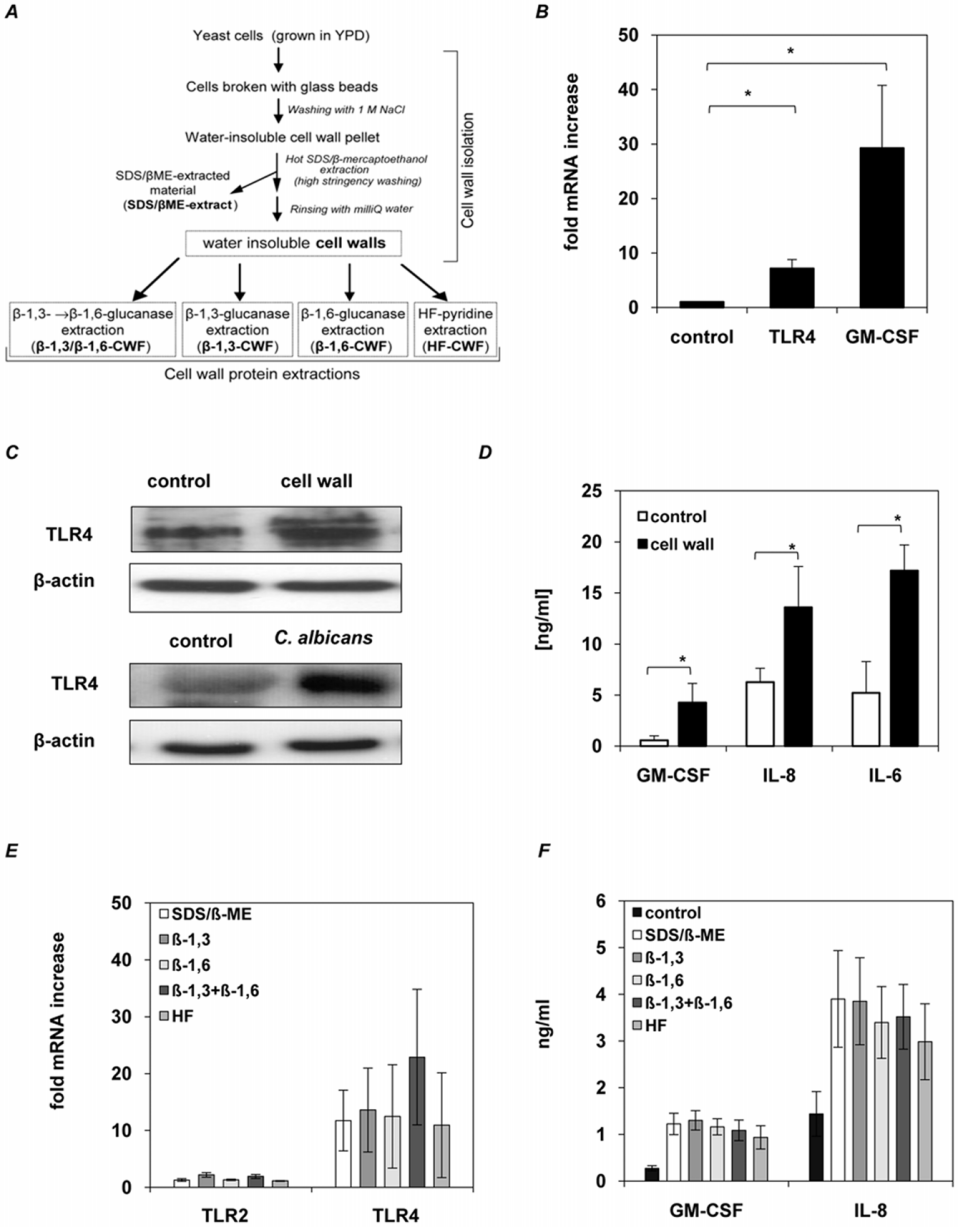
Effector responses of human oral epithelial cells to *C. albicans* cell wall extracts. Epithelial immune response is triggered by *C. albicans* cell wall structure. (**A**) For cell wall extracts, *C. albicans* yeast cells were broken with glass beads and insoluble cell walls were collected by centrifugation. Cell wall pellets were heated in SDS/β-Me extraction buffer to remove non- covalently bound protein material (SDS/β-ME-CWF) from the cell wall fragments. Afterwards covalently bound cell wall proteins were extracted from washed and dialyzed water insoluble SDS-treated cell walls with either HF-pyridine (HF-CWF), endo-β-1,3-glucanase (β-1,3-CWF), endo-β-1,6-glucanase (β-1,6-CWF) or endo-β-1,3-glucanase and endo-β-1,6-glucanase (β-1,3/β-1,6-CWF). Cell wall extracts isolated from 1×10^8^ *Candida* cells (MOI = 100) were used to stimulate human epithelial cells (1×10^6^ cells) for 24 h. (**B**) *C. albicans* walls induced TLR4 and GM-CSF mRNA up regulation in epithelial cells as determined by quantitative RT-PCR. Data are given as relative mRNA expression compared to mRNA expression of PBS-treated control cells (control = 1.0). (**C**) Immunoblot of induced TLR4 protein levels in epithelial cells after 12 h incubation with viable *C. albicans* (MOI = 100) and 24 h incubation with cell walls. (**D**) Amount of GM-CSF, IL-8 and IL-6 in the culture supernatants of cell wall-stimulated epithelial cells and PBS-treated control cells as measured by ELISA. (**E**) CWF-induced TLR4 mRNA up regulation in epithelial cells as determined by quantitative RT-PCR. Data are given as relative mRNA expression compared to mRNA expression of PBS-treated control cells (control = 1.0). (**F**) Amount of GM-CSF and IL-8 in the culture supernatants of CWF- stimulated epithelial cells and PBS-treated control cells, as measured by ELISA. (**B, D** to **F)**, *n* = 4 (± *SEM*), * p<*0.05*, 2-tailed paired Student’s *t* test and (**C)** representative data of three independent experiments.

First we generally investigated the ability of *C. albicans* to activate epithelial cytokine response by stimulating TR146 oral epithelial cells for 24 h with either viable yeast cells, able to switch from yeast to hyphal growth, heat-treated yeast cells and hyphae as well as UV treated yeast cells to rule out possible effects on the cell wall by the method used for fungal killing. Increased GM-CSF (Fig. 1A), IL-6 and IL-8 (data not shown) secretion was observed for all tested samples and significant differences appeared between viable and heat-treated cells, whereas no significant differences in GM-CSF induction could be observed between viable and UV treated cells or cells in yeast and hyphal growth form (Fig. 1A). The observed increased cytokine induction by heat-treated cells but not UV treated cells compared to viable cells is most likely due to loss of membrane integrity, release of intracellular content and changes in cell wall structure that further stimulates cytokine induction in epithelial cells (Fig. 1A). GM-CSF induction was dependent on the number of yeast cells used for stimulation as low numbers of yeast cells induced a higher secretion of GM-CSF (Fig. 1B), IL-6 and IL-8 (data not shown) as compared to samples stimulated with higher yeast cell numbers. These findings indicate, that the induction of cytokine secretion by *C. albicans* in epithelial cells dependents on the amount of stimuli, but is independent from the morphological change of *C. albicans* from yeast to hyphal growth. As the fungal cell wall is known to possess immune activatory components, water insoluble cell walls of *C. albicans* were isolated as described in Figure 2A and used to stimulate oral epithelial cells. Gene expression analysis of pattern recognition receptors related to myeloid *C. albicans* recognition, including TLR2, TLR4, TLR6, TLR9, Dectin-1 and mannose receptor only showed an increased level of TLR4 mRNA (Fig. 2B and Fig. S1) and protein (Fig. 2C), that was also observed for viable *C. albicans* (Fig. 2C). Furthermore, oral epithelial cells secreted increased amounts of GM-CSF, IL-6 and IL-8 (Fig. 2D). To identify whether a certain class of cell wall protein was responsible for epithelial responses, we repeated the experiments with four additional but better-defined cell wall protein fractions (CWFs) and with the SDS/β-mercaptoethanol extract (Figure 2A and Table 2). However, identical results were observed with all fractions for induced TLR4 mRNA levels (Fig. 2E), and for GM-CSF and IL-8 secretion (Fig. 2F). Notably, mRNA levels for TLR2 were unaffected (Fig. 2E), demonstrating specificity to TLR4.

**Table 2:** Content of the cell wall fractions.

cell wall fraction	extraction method(enzyme/substances)	putative content
SDS/β-ME	SDS/β-ME-buffer	non-covalently bound proteins; membrane proteins; cytosolic proteins; PIR-proteins; GPI-proteins
β-1,3	endo-β-1,3-glucanase	GPI-proteins- β-1,6-glucan complexes; PIR-proteins; remnants of β-1,3-glucan chains
β-1,6	endo-β-1,6-glucanase	GPI-proteins
β-1,3/β-1,6	endo-β-1,3-glucanase and endo-β-1,6-glucanase	PIR-proteins; GPI-proteins
HF	hydrofluoric acid-pyridine	GPI-proteins without phosphomannan (part of the *N*-glycan chains)

mannoproteins are marked in **bold.**

GPI: glycosylphosphatidylinnositol; PIR: protein with internal repeats;

SDS: sodiumdodecylsulfate; HF: hydrofluoric acid; β-ME: mercaptoethanol.

Extensive studies ruled out any contamination issues with SDS (<0.001% (w/v)), β-ME (undetectable) or LPS (<100 pg/ml). The data indicate that epithelial cell stimulation is due to common components present in all CWFs. Given this, all future stimulation experiments were performed using water insoluble (SDS/β-ME-treated) cell walls.

### Induction of Epithelial Cytokines by *C. albicans* Cell Walls is Independent of TLR2, TLR4, MR or Dectin-1

**Figure 3:**
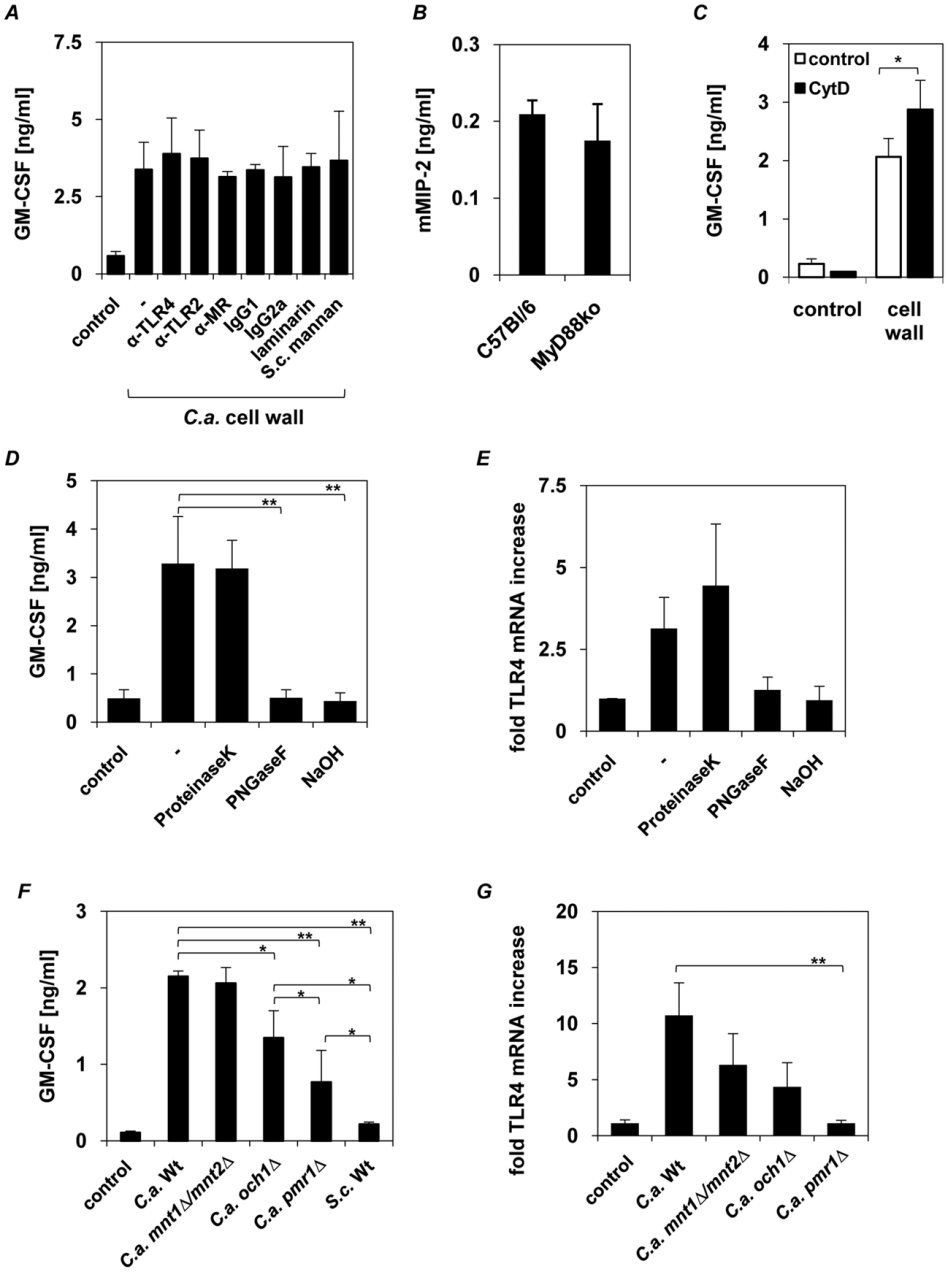
Epithelial cytokine induction is independent of TLR2, TLR4, dectin-1 and MR. Human epithelial cells (1×10^6^) were pre-incubated with (**A**) 10 µg/ml anti-TLR2, anti-TLR4, anti-MR antibodies, laminarin (100 µg/ml) or *S. cerevisiae* mannan (40 µg/ml) 2 h before epithelial cells were stimulated with *C. albicans* walls (1×10^8^) for 24 h. (**B**) Oral epithelial cells (3×10^5^) isolated from wild-type and MyD88−/− mice were incubated for 24 h with isolated walls (3×10^7^). (**C**) Human epithelial cells (1×10^6^) were incubated with 5 µM cytochalasin D for 30 min prior stimulation with *C. albicans* walls (1×10^8^) for 24 h (**D** and **E**) Cell wall mannoproteins were deproteinized by incubating *C. albicans* walls (1×10^8^) with proteinase K or deglycosylated by PNGaseF digestion (cleaves *N-*glycosylation) or NaOH treatment (alkaline β-elimination reduces *O*-glycosylation). Human epithelial cells (1×10^6^) were incubated for 24 h with isolated walls (as positive control) or proteinase K-, PNGaseF- and NaOH-treated walls. (**F** and **G**) Epithelial cells (1×10^6^) were incubated for 24 h with cell walls (1×10^8^) isolated from *C. albicans* wild type (SC5314), *N-*glycosylation (*och1Δ*), *O*-glycosylation (*mnt1Δ/mnt2Δ*), *N−/O*-glycosylation (*pmr1Δ*) mutant strains or non- pathogenic *S. cerevisiae*. Human GM-CSF and mouse MIP-2 were quantified by ELISA. TLR4 mRNA up regulation in epithelial cells was determined by quantitative RT-PCR. Data are given as relative mRNA expression compared to mRNA expression of PBS-treated control cells (control = 1.0). (**A–G**), *n* = 3 (± *SEM*), * p<0.05, **p<0.01, 2-tailed paired Student’s *t* test.

Our data indicated that *C. albicans* CWFs mediate the upregulation of epithelial TLR4 and cytokine production. However, the receptors through which these innate responses are initiated are unknown. Given that TLR4, TLR2, dectin-1 and MR have been shown to mediate the *C. albicans*-induced cytokine response in myeloid cells [6], we pre-incubated oral epithelial cells with neutralizing TLR2, TLR4 and MR monoclonal antibodies, or with *S. cerevisiae* mannan [22] or laminarin (a soluble β-1,3-glucan from algae), prior to stimulation with water insoluble *C. albicans* cell walls to block possible cell wall-receptor interactions. Laminarin interacts with dectin-1, internalizing the receptor, thereby inhibiting dectin-1 activation by *C. albicans* β-glucan in macrophages [23]. Isotype-matched antibodies were included as negative controls. Introduction of anti-TLR2, anti-TLR4, anti-MR antibodies, mannan or laminarin did not alter the increased secretion of GM-CSF (Fig. 3A), IL-6, or IL-8 (data not shown) 24 h after exposure to *C. albicans* walls as compared with the isotype-matched controls. *S. cerevisiae* mannan and laminarin blocking capacity as well as the functional activity of the anti-TLR2, anti-TLR4 and anti-MR antibodies was confirmed as previously demonstrated [24] by pre-incubating human PBMCs with these substances followed by stimulation with heat-killed yeast or LPS, which resulted in significant reduction of GM-CSF cytokine secretion (Fig. S2). Results for TLR/MyD88-independent cytokine induction in epithelial cells were confirmed by stimulating primary buccal epithelial cells from wild type and MyD88-deficient mice with *C. albicans* cell walls. Induced mMIP-2 secretion does not differ in MyD88-deficient cells compared the amount induced in wild type cells (Fig. 3B).

As MR and Dectin-1 are classical phagocytic receptors, we treated the epithelial cells with the endocytosis inhibitor cytochalasin D prior stimulation with *C. albicans* cell wall, to test whether uptake is necessary or intracellular PRRs are involved in triggering the observed cytokine induction. Interestingly, blocking the up-take by epithelial cells lead to an increased GM-CSF production compared to untreated cells (Fig. 3C).

Therefore, induction of the epithelial cytokine response by the *C. albicans* cell walls is independent of TLR2, TLR4, MR or dectin-1 activation, but most likely triggered by a receptor located on the epithelial surface.

#### Glycosylation of cell wall mannoproteins is critical for inducing epithelial immune responses

Given that epithelial responses are induced by components common to all the *C. albicans* CWFs tested (Fig. 2), we hypothesized that the glycosylated regions of the cell wall-associated mannoproteins present in all these CWFs might be responsible for oral epithelial cell stimulation (Table 2). To test this hypothesis, we deglycosylated the cell wall mannoproteins by treatment with PNGaseF (removal of *N*-glycosylation) and/or NaOH (removal of *O- and* partially *N*-glycosylation) and compared the immune response with (i) untreated cell walls and (ii) cell walls after digestion of the peptide cores by proteinase K. Deproteinized and untreated walls both resulted in the normal induction of GM-CSF (Fig. 3D), IL-6, IL-8 secretion (data not shown) and TLR4 mRNA expression (Fig. 3E), while removal of *N*- or *O*-glycans from the cell wall proteins abolished cytokine induction (Fig. 3D) and TLR4 mRNA increase (Fig. 3E). This indicates that both the *N*- and *O*-linked mannosyl chains contribute to immune activation of mucosal epithelial cells.

To verify these results, we isolated cell walls from three *C. albicans* glycosylation mutants (*och1Δ*, *mnt1/mnt2Δ*, *pmr1Δ*) and the non-pathogenic fungus *Saccharomyces cerevisiae* (overview of strains and phenotypes given in Table 1) [6], [25], [26], [27]. The *och1Δ* mutant has a defect in the outer, branched *N*-linked glycans, the *mnt1/mnt2Δ* mutant lacks the four terminal *O*-linked α_1,2_-mannosyl residues on the short *O*-glycans, and the *pmr1Δ* mutant has defects in both *N*- and *O*-mannosylations. Whereas the cell walls from the *mnt1/mnt2Δ O*-mannosylation mutant induced same levels of GM-CSF in comparison with wild-type cell walls (Fig. 3F), cell walls from the *och1Δ N*-mannosylation mutant showed significantly reduced GM-CSF induction. The loss of both, *N*- and *O*-mannosyl residues, as represented in the *pmr1Δ* mutant, reduced GM-CSF induction significantly compared to wild-type and *och1Δ* mutant (Fig. 3F), whereas the *S. cerevisiae* cell wall failed to induce any cytokine response. A similar pattern was observed for TLR4 mRNA induction (Fig. 3G). The data demonstrate that both *N*- and *O*-mannosyl residues activate mucosal epithelial cell responses, but with a predominant role for *N*-mannosyl residues.

#### Inhibition of epithelial proliferation followed by epithelial apoptosis

**Figure 4:**
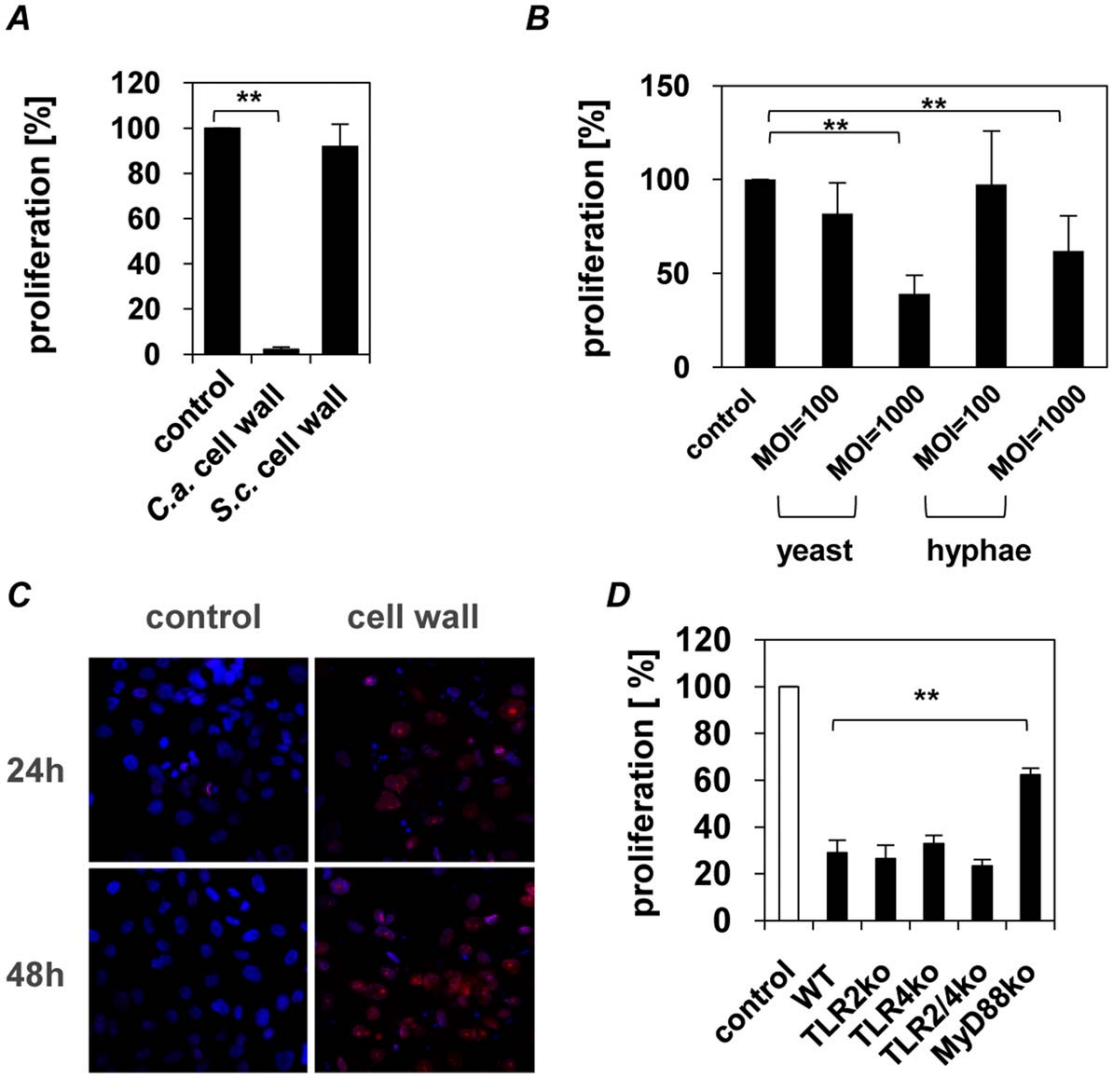
*C. albicans* cell wall has MyD88-dependent anti-proliferative effect on mucosal epithelial cells. (**A**). Proliferation rates were quantified after 24 h by [^3^H]-thymidine assay. [^3^H]-thymidine (1 µCi/ml) was added 6 h after starting the incubation of human epithelial cells (1×10^4^) with *C. albicans* or *S. cerevisiae* walls (1×10^6^), which was continued for 18 h. (**B**) Proliferation rate of human epithelial cells (1×10^4^) incubated with increasing numbers of heat-killed *C. albicans* yeast or hyphae (1×10^6^ and 1×10^7^, MOI = 100 to 1000). (**C**) Confocal laser scanning microscopy of the cell cycle inhibitor p27^kip1^ was performed after 24 h and 48 h of incubated human epithelial cells (1×10^5^) with *C. albicans* cell walls (1×10^7^) or PBS as control (cell nuclei = blue, p27^kip1^ =  red). Note the increase and nuclear accumulation of the cell cycle inhibitor p27^kip1^ in the wall-treated cells. (**D**) Oral epithelial cells (3×10^5^) isolated from wild-type, TLR2−/−, TLR4−/−, TLR2/4−/− and MyD88−/− mice were incubated for 24 h with isolated walls (3×10^7^). Proliferation of murine oral epithelial cells from was measured by BrdU-ELISA. (**A,** **B** and **D**) *n* = 4 (± *SEM*), ** p<*0.01*, 2-tailed paired Student’s *t* test and (**C**) Pictures shown are representative of three independent experiments.

In cell culture we observed that the pH values of the media exposed to all *C. albicans* CWFs did not change while untreated epithelial cells induced an acidic milieu. This prompted us to hypothesize that the cell walls may inhibit cell proliferation. To test this, [^3^H]-thymidine incorporation into epithelial cell DNA was analyzed after exposure to isolated cell walls. Epithelial cells that were exposed for 24 h to cell walls showed a diminished [^3^H]-thymidine incorporation with up to 90% of cells being non-proliferative (Fig. 4A). Cell walls isolated from the non-pathogenic fungi *S. cerevisiae* failed to inhibit epithelial proliferation (Fig. 4A), indicating specificity to *C. albicans* cell walls. Heat-killed yeast cells or hyphae do not alter epithelial proliferation in the same concentration used for isolated cell walls, but do significantly inhibit proliferation in higher concentrations, but independent of the morphology (Fig. 4B).

To define the mechanism of the anti-proliferative effects induced by exposure to *C. albicans* cell walls, the effect on critical molecular events known to regulate the cell cycle and the apoptotic machinery was assessed. Inhibition of epithelial proliferation was associated with a strong accumulation of the cell cycle inhibitor p27^kip1^ inside the nucleus as demonstrated by confocal laser scanning microscopy (Fig. 4C). The cyclin-dependent kinase p27^kip1^, whose major target is the cyclinE/CDK2 complex, governs cell cycle transition from late G1 to S phase, and has also been implicated in the regulation of apoptosis, differentiation, and in the cell response to inflammatory stimuli [28], [29].

Recent reports suggest that in addition to their role in antimicrobial defense, TLR family members are involved in regulating cell proliferation and tissue repair [30], [31]. To analyze the role of epithelial TLR2, TLR4, dectin-1 and MR in proliferation inhibition we pre-incubated TR146 oral epithelial cells with neutralizing anti-TLR2, -TLR4 and -MR monoclonal antibodies or laminarin and *S. cerevisiae* mannan (including isotype-matched antibodies as negative controls). Inhibition of cell proliferation was not influenced by any of these treatments (data not shown). The data was confirmed with primary murine buccal epithelial cells derived from TLR-deficient mice after stimulation with cell walls. Proliferation rates of TLR2^−/−^, TLR4^−/−^, and TLR2^−/−/^TLR4^−/−^ epithelial cells were similar to epithelial cells derived from wild-type mice (Fig. 4D). However, interestingly, the proliferation rates of MyD88−/− epithelial cells were significantly less affected demonstrating a potential role for MyD88 and MyD88-dependent signaling pathways in proliferation inhibition (Fig. 4D).

**Figure 5:**
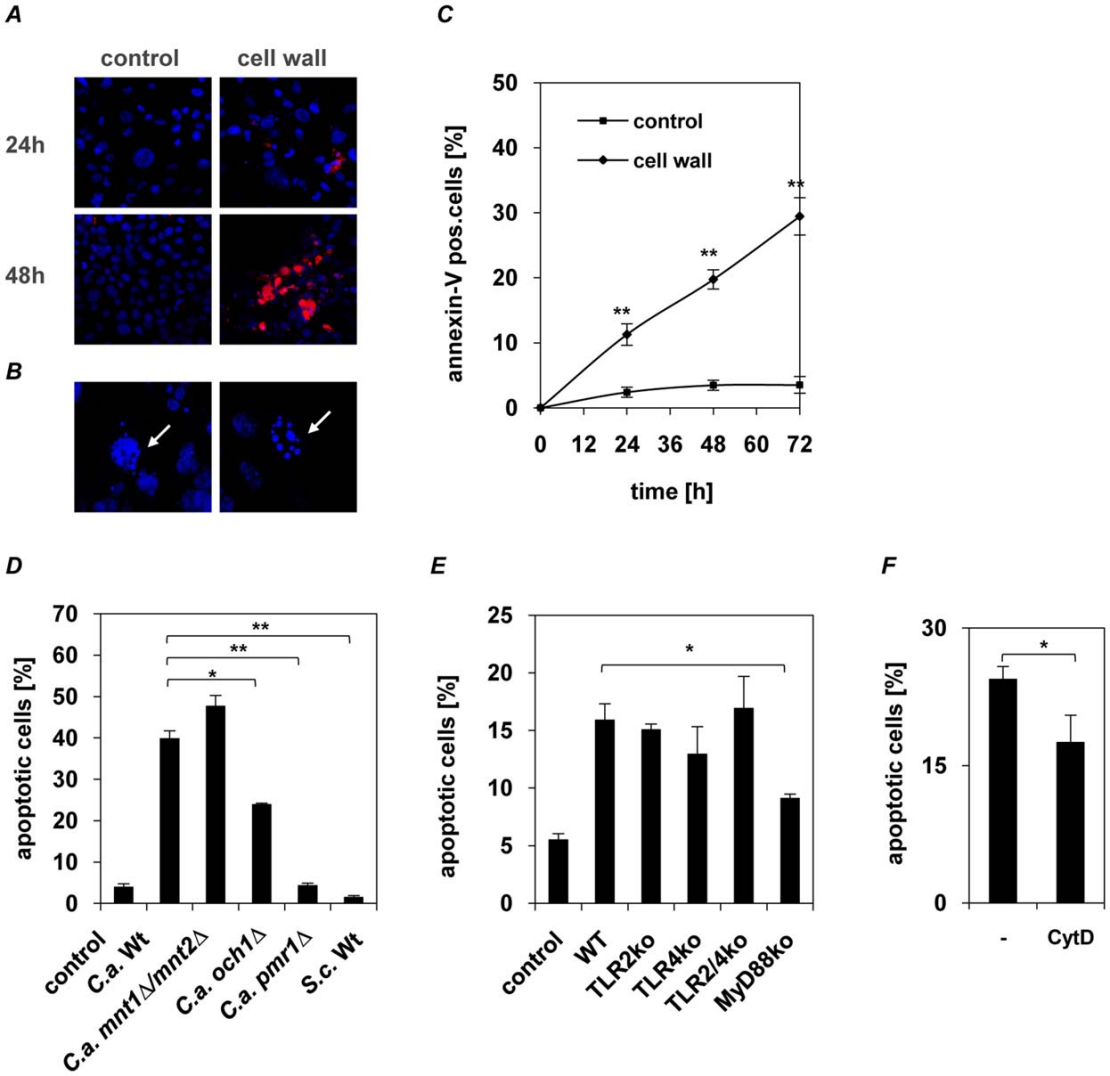
*C. albicans* cell wall glycosylation-dependent apoptosis induction in oral epithelial cells. (**A** and **B**) Confocal laser scanning microscopy of the active caspase-3 (***red***) and nuclei (***blue***) in cell wall-stimulated human epithelial cells or PBS-treated control after 24 and 48 h of incubation. Typical morphological apoptotic characteristics observed after 24 h stimulation with cell walls with nuclear condensation (right) and apoptotic body formation (left) are indicated (**B**, ***white arrows***). (**C**) Apoptosis and necrosis of epithelial cells was analyzed using Annexin-V/PI staining. No large increase of necrotic (PI single stained) cells but a clear increase of apoptosis with ∼30% apoptotic epithelial cells was observed after 72 h of incubation with *C. albicans* cell walls. (**D**) Human epithelial cells (1×10^6^) were incubated with cell walls isolated from *C. albicans* wild-type (SC5314), *N*-glycosylation (*och1Δ*), *O-*glycosylation (*mnt1Δ/mnt2Δ*), *N−/O*-glycosylation (*pmr1Δ*) mutant strains or *S. cerevisiae*. Walls isolated from the *N*-glycosylation (*och1Δ*) induced significantly less apoptosis compared to wild-type walls, whereas walls from the *N−/O*-glycosylation mutant (*pmr1Δ*) and *S. cerevisiae* completely failed to induce apoptosis. (**E**) Oral epithelial cells (1×10^6^) isolated from wild-type, TLR2−/−, TLR4−/−, TLR2/4−/− and MyD88−/− mice were incubated with isolated walls from *C. albicans* wild-type (SC5314). Apoptosis was analyzed by flow cytometry with Annexin/PI-staining after 72 h. Loss of MyD88-mediated signalling significantly reduced apoptosis induction. (**F**) Human epithelial cells (1×10^6^) were incubated with 5 µM cytochalasin D for 30 min prior stimulation with *C. albicans* walls (1×10^8^) and apoptosis was analyzed by flow cytometry with Annexin/PI-staining. Endocytosis blocking significantly reduced cell wall induced apoptosis. (**A** and **B**) Pictures shown are representative of three independent experiments. (**C to F**) *n* = 3 (± *SEM*), *p<*0.05*, **p<*0.01*, 2-tailed paired Student’s *t* test.

Time-series analysis showed that inhibition of proliferation is followed by an increased activation of the cysteine protease caspase-3 (Fig. 5A), which plays a crucial role in apoptotic pathways by cleaving a variety of key cellular proteins. Furthermore, an increase in apoptotic cells was observed by typical morphology (Fig. 5B) and flow cytometry analysis of annexin V expression (Fig. 5C).

#### Induction of epithelial apoptosis critically depends on intact cell wall glycosylation

To investigate the role of cell wall protein glycosylation in inducing epithelial apoptosis, isolated cell walls from the *C. albicans* wild-type strain, *N-* and *O-*glycosylation mutants and *S. cerevisiae* (Table 1) were used. Loss of *O*-mannosyl residues (*mnt1/mnt2Δ*) marginally increased apoptosis induction, while loss of *N*- mannosyl residues (*och1Δ*) significantly reduced apoptosis induction (p<0.05). However, loss of both *N*- and *O*-mannosyl residues (*pmr1Δ*) totally abolished apoptosis induction (p<0.01) (Fig. 5D).

Furthermore, apoptosis induction was not influenced by pre-incubation of epithelial cells with neutralizing anti-TLR2, -TLR4 and -MR monoclonal antibodies or laminarin and *S. cerevisiae* mannan (data not shown). Only apoptosis in primary murine epithelial cells from MyD88-deficient mice were significantly reduced (Fig. 5E) compared with wild-type, TLR2^−/−^, TLR4^−/−^, and TLR2^−/−/^TLR4^−/−^ epithelial cells. Furthermore endocytosis blocking, does not only influence the cell wall induced cytokine secretion (Fig. 3G), also apoptosis induction is reduced in epithelial cells unable to take up *C. albicans* cell wall particles (Fig. 5F).

Our data demonstrate that glycosylation of *C. albicans* cell wall proteins, particularly *N*-glycosylation, is critical for inducing apoptosis in epithelial cells, partially mediated through MyD88.

#### Early apoptosis induction during oral *Candida* infection depends on intact cell wall protein glycosylation

**Figure 6:**
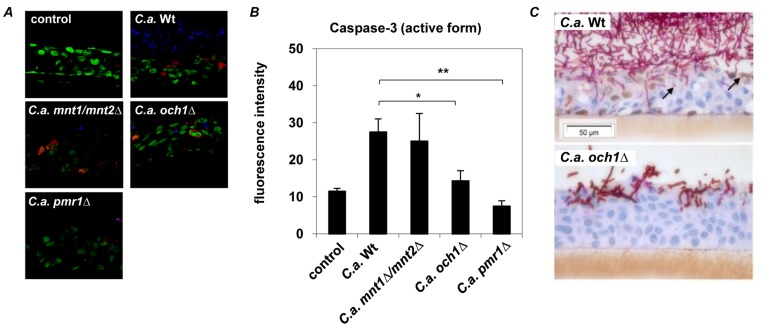
Cleaved (activated) caspase-3 expression in *C. albicans* infected oral RHE. Oral RHE was infected with 2×10^6^ yeast cells from either *C. albicans* wild-type (SC5314), *N*-glycosylation (*och1Δ*), *O-*glycosylation (*mnt1Δ/mnt2Δ*) or *N−/O*-glycosylation (*pmr1Δ*) mutant strains for 4 h or 24 h. (**A**) Confocal microscopy of active caspase-3 expression (***red***) in RHEs after 4 h showed no caspase-3 induction in uninfected () or *N−/O*-glycosylation (*pmr1Δ*) mutant infected epithelium, whereas infection with *C. albicans* wild-type or *O-*glycosylation (*mnt1Δ/mnt2Δ*) mutant strain increased caspase-3 activation after 4 h of incubation. In *N*-glycosylation (*och1Δ*) mutant infected RHE reduced amounts of caspase-3 were observed. (**B**) Significantly reduced mean fluorescence intensity was observed in *O-*glycosylation (*mnt1Δ/mnt2Δ*) and *N−/O*-glycosylation (*pmr1Δ*) mutant infected RHEs compared to wild-type infected epithelium. (**C**) Cleaved caspase-3 expression (brown staining) is seen after 24 h in the nuclei of RHE cells adjacent to invading fungal hyphae and in apoptotic bodies (black arrows) in SC5314 but not Δ*och1*infected cultures. (**A**) Pictures shown are representative of three independent experiments; nuclei of epithelial cells are stained in ***green*** and *C. albicans* in ***blue***. (**B**) Mean results of 10 analysed slides of three independent experiments, *n* = 3 (± *SEM*), *p<0.05, **p<*0.01*, 2-tailed paired Student’s *t* test.

To confirm our findings in context of an *in vivo* resembling situation, we used an established *in vitro* model of oral candidosis. Reconstituted human epithelium (RHE) was infected with *C. albicans* wild-type strain or glycosylation mutants (*och1Δ*, *mnt1/mnt2Δ*, *pmr1Δ)* for 4 h or 24 h (Fig. 6). *C. albicans* wild-type strain significantly induced caspase-3 (activated form) after 4 h of infection in the oral epithelium (Fig. 6A and B), whereas epithelial damage analyzed by LDH release due to invading hyphae could not be detected at this early time point. *O*-glycosylation (*mnt1/mnt2Δ*) mutant induced equal amounts of activated caspase-3 compared to wild-type strain after 4 h, whereas the *N*-glycosylation mutant (*och1Δ*) induced significantly less caspase-3 activation and the loss of *N*- and *O*- glycosylation (*pmr1Δ*) totally abolished caspase-3 activation (Fig. 6A and B). After 24 h infection activated caspase-3 expression could be observed in epithelial cells adjacent to invading hyphae and in apoptotic bodies of wild-type infected RHE, but not in *N*-glycosylation mutant (*och1Δ*) infected RHE (Fig. 6C).

## Discussion

The *C. albicans* cell wall is the key structure mediating host-fungal interactions. Several studies have demonstrated the importance of *C. albicans* cell wall components in activating dendritic cells [32], [33], T-cells [34], [35] and macrophages [6], which may result in protective immunity against systemic and localized candidiasis. However, the interaction between the fungal cell wall and epithelial cells has been largely neglected and our understanding of the effect of these interactions on epithelial biology and immunity is elementary.

Given the importance of local immunity in controlling mucosal *Candida* infections, we characterized the ability of *C. albicans* cell wall components to activate innate immune responses in epithelial cells. We demonstrate the importance of *C. albicans* cell wall glycans in mediating fungal-epithelial cell interactions and present new findings concerning the role of the outer fungal cell wall layer of highly glycosylated mannoproteins in mediating epithelial immune responses and in inducing apoptosis, which may in turn promote fungal virulence.

We first examined the ability of *C. albicans*, the fungal cell wall and cell wall-derived protein fractions to trigger an epithelial pro-inflammatory cytokine (GM-CSF, IL-6, IL-8) and pattern recognition receptor (PRR) induction, namely TLR4. Our data indicate that the glycan moieties of cell surface mannoproteins are the principal components inducing cytokine responses in oral epithelial cells. This immune stimulatory activity of glycan moieties is consistent with studies in human dendritic cells [32], [33] human mononuclear cells [6] and epithelial cells [36], Furthermore, we demonstrate that *N*-mannosylation is more critical for cytokine induction than *O*-mannosylation, but both are required since only the walls isolated from the *pmr1Δ* mutant (deficient in both *N*- and *O*-mannosylation) failed to induce any cytokine responses. Our data are supported by a recently published study demonstrating the contribution of *N*- and *O*- glycosylation to epithelial activation and cytokine responses [16]. However, it is in contrast with another study that did not observe epithelial cytokine activation with purified *C. albicans N*- and *O*-glycans [37]. In the latter study the authors hypothesized that the lack of cytokine response may be because the purified polysaccharide components may differ from their steric presentation in the complex structure of a viable cell wall. Our data using viable and heat-killed *C. albicans* yeast cells and hyphae as well as the purified cell walls from *C. albicans* cells support this view and indicate the lack of induction by purified *N*- and *O*- glycans was likely due to the loss of structural and/or constitutional properties due to the extraction process. Moreover, this hypothesis is supported by observations that size and solubility of the recognized structure is important for receptor activation as demonstrated for β-glucan/dectin-1 [38].

In addition to the importance of protein glycosylation in inducing cytokine responses, our data indicate no role for β-glucan or chitin in mediating epithelial responses to *C. albicans* cell walls. This is consistent with several recent studies observing no role for dectin-1 in epithelial cell activation [16], [37].

*C. albicans* recognition by myeloid cells is mediated by several different receptors, all recognizing different molecules of the *Candida* cell wall. For example, TLR4 recognizes *O*-linked mannans, the macrophage mannose receptor recognizes *N*-linked mannans and β-glucan is recognized by dectin-1. This recognition is responsible for the majority of the cytokine stimulating activity of *C. albicans* in myeloid cells [6]. In contrast to myeloid cells, we report that cytokine induction in epithelial cells is independent of TLR2, TLR4, MR or dectin-1. Our data is supported by previous studies demonstrating that knockdown of TLR2 and TLR4 expression with siRNA also did not influence the cytokine profile induced by *C. albicans* in oral epithelial cells [37]. Nevertheless, using cell walls from *C. albicans* cell wall glycosylation mutants we demonstrate that cytokine induction in epithelial cells is triggered by *N*- and *O*- glycans, but the epithelial receptors involved remain unidentified.

Exposure of epithelial cells to glycosylated mannoproteins induced cell cycle arrest and apoptosis. Induction of the anti-proliferative and apoptotic cell machinery was also confirmed in primary human (data not shown) and murine epithelial cells, demonstrating universal biological relevance of the observed mechanism. We also confirmed our data by RT-PCR studies demonstrating an increase in Bax mRNA and decrease in Bcl-2 mRNA (data not shown). Bax is a pro-apoptotic member of the Bcl-2 family and Bcl-2 is an anti-apoptotic member, and both are involved in the regulation of genetically programmed cell death. Using cell wall preparations of *C. albicans N*-, *O*- and *N*−/*O*-glycosylation mutants and pretreated wild-type preparations we found a more pronounced role in apoptosis induction for the *N*-glycans rather than *O*-glycans. However, both *N*- and *O*-glycosylation is involved in apoptosis induction since only the cell wall of the double deficient mutant (deficient in both *N*- and *O*-glycosylation) failed to induce apoptosis. The outermost cell wall layer of *C. albicans* is densely packed with surface proteins that are highly glycosylated. Although some studies suggest a minor role for this mannoprotein layer in stimulating systemic immunity [7], a number of other studies indicate a prominent role for the mannosylation in systemic as well as local *Candida* infections [6], [39], [40].

Our novel findings may provide insight into an important process in *C. albicans* pathogenesis since cell death, particularly of epithelial cells, might favor invasion of mucosal surfaces by the fungus. To date, *C. albicans-*induced apoptosis has been described in several human and murine cells [41], [42], [43], [44], [45], [46], [47]. *C*. *albicans*-induced apoptosis of murine macrophages has also been described [42], but was induced only by viable and not heat-killed fungal cells, indicating that macrophage apoptosis might depend upon a factor actively released by *C*. *albicans*. Likewise, a recent study demonstrated that viable *C. albicans* cells were required to induce early apoptosis in oral epithelial cells followed by late necrosis [48]. The fungal agents activating these apoptotic processes remain unclear although host cell internalization of *C. albicans* may be required. Irrespective, we provide strong evidence that cell wall glycans are the inducing agents of epithelial cell apoptosis and proliferation arrest by viable *C. albicans*, with a more profound role for *N*-glycans. However, as with cytokine responses, both *N*- and *O*-glycans are required since induction of apoptosis was only abolished when water insoluble cell wall from the *pmr1Δ* mutant was used. Given our data and the fact that β-glucans and chitin are effectively masked by glycosylated mannoproteins in the outer layer of viable cell walls [23], it is plausible that cell wall glycans might also be the inducing agents of apoptosis in macrophages [42]. This hypothesis is supported by recent work using murine macrophages, which demonstrated the reduced ability of *C. albicans* cell wall glycosylation mutants to kill macrophages after phagocytosis, via a process independent of hyphae formation [39].

Activation of TLRs is known to have pro-apoptotic effects, with the adapter protein MyD88 being critically involved in this mechanism. MyD88 is able to interact with FADD (Fas-associated protein with death domain) to induce caspase-8 activation and ultimately apoptosis induction [29]. Therefore, a further novel finding is that induction of epithelial apoptosis by *C. albicans* appears to be independent of TLR2, TLR4 or MR. This was demonstrated by antibody blocking studies and supported by data using epithelial cells from TLR2^−/−^, TLR4^−/−^ and TLR2^−/−/^4^−/−^ mice. However, inhibition of epithelial proliferation by *C. albicans*, while also independent of TLR2, TLR4 or MR, was dependent to some extent on MyD88 as oral epithelial cells from MyD88^−/−^ mice showed significantly higher proliferation rates in the presence of *C. albicans* cell walls and less apoptosis induction. Therefore, our data suggest that MyD88 is involved in epithelial proliferation and apoptosis, which is likely mediated through signaling pathway interplay rather than *C. albicans* cell wall interaction with a specific pattern recognition receptor.

In intestinal mucosa, LPS-induced TLR4 activation and enhanced NF-κB induction leads to increased apoptosis in enterocytes of new born mice, but not adult mice. Due to different expression levels of TLR4 in newborn and adult mice, TLR4 levels may be important for homeostatic versus pathological role [49]. We previously showed that activation of neutrophils protects epithelial tissues against fungal invasion via epithelial TLR4 up regulation [24]. Although the precise role of TLR4 in mucosal protection against fungal infection is still unclear, our data indicate that the observed increase in TLR4 expression of oral epithelial cells through glycosylated regions of *C. albicans* cell wall proteins may be more critical in inducing subsequent protective responses and to maintain epithelial homeostasis rather than initiating inflammation. Further studies are clearly warranted to identify the receptors and pathway interactions responsible for inducing *C. albicans*-mediated apoptosis in epithelial cells.

In conclusion, *N-* and *O-*glycosylation of *C. albicans* cell wall proteins is not only a potent stimulus of epithelial innate responses but is also critical for the regulation of epithelial proliferation and apoptotic mechanisms. This demonstrates the potential importance of these fungal surface moieties in epithelial-fungal interactions and in promoting fungal pathogenesis.

## Supporting Information

Figure S1 **mRNA expression profile of epithelial cells challenged with** ***C. albicans.*** Cell wall isolated from 1×10^8^ *Candida* cells (MOI = 100) were used to stimulate human epithelial cells (1×10^6^ cells) for 24 h and induced mRNA expression was determined by quantitative RT-PCR. Data are given as relative mRNA expression compared to mRNA expression of PBS-treated control cells (control = 1.0). *n* = 4 (± *SEM*), * p<*0.05*, 2-tailed paired Student’s *t* test.
(TIF)

Figure S2 **Positive blocking effect of antibodies and carbohydrates.** Human PBMCs (1×10^6^) were pre-incubated with 10 µg/ml anti-TLR2, anti-TLR4, anti-MR antibodies, laminarin (100 µg/ml) or *S. cerevisiae* mannan (40 µg/ml) 1 h before cells were stimulated with heat-treated *C. albicans* (1×10^8^) for 24 h or LPS (100 ng/ml). GM-CSF was quantified by ELISA. *n* = 3 (± *SEM*), * p<0.05, **p<0.01, 2-tailed paired Student’s *t* test.
(TIF)
