# Supplementary figures and images for: Glycosylation of Candida albicans Cell Wall Proteins Is Critical for Induction of Innate Immune Responses and Apoptosis of Epithelial Cells
Source: PLoS One. 2012 Nov 30;7(11):e50518. doi: 10.1371/journal.pone.0050518 (PMC3511564; doi:10.1371/journal.pone.0050518)

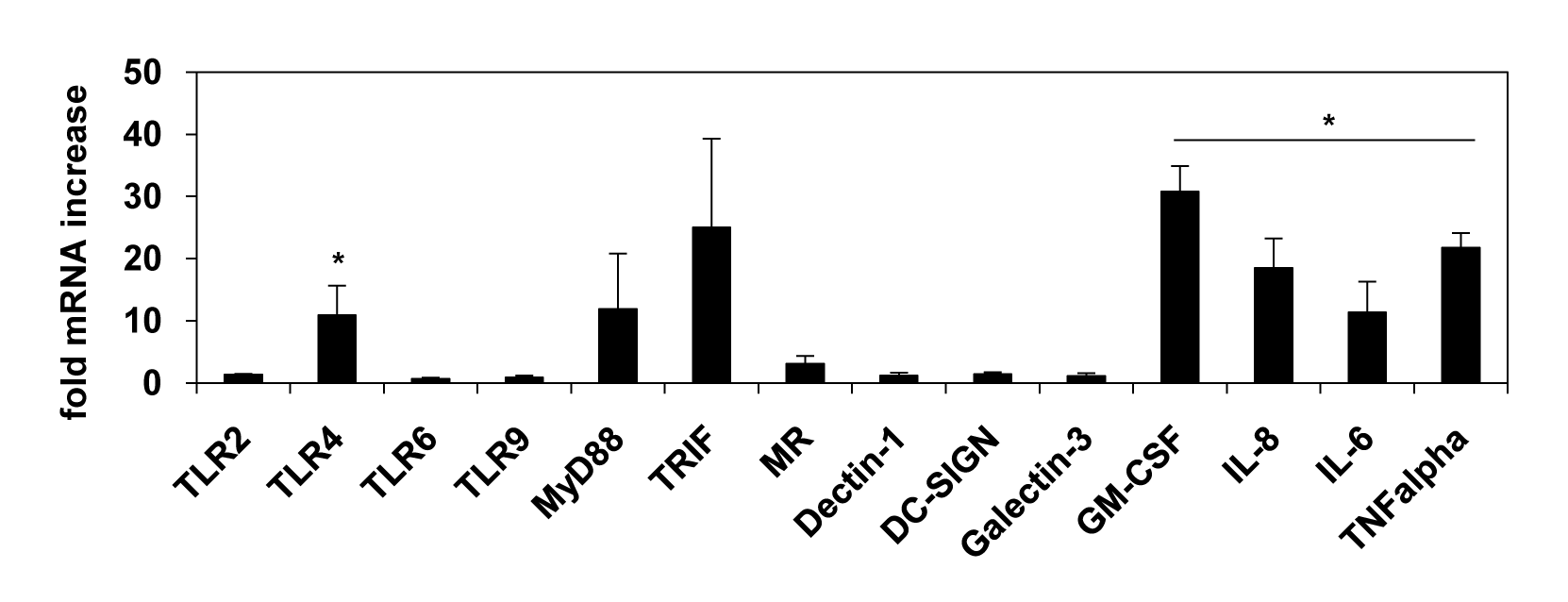

Supplement: Figure S1 — mRNA expression profile of epithelial cells challenged with C. albicans. Cell wall isolated from 1×10^8^ Candida cells (MOI = 100) were used to stimulate human epithelial cells (1×10^6^ cells) for 24 h and induced mRNA expression was determined by quantitative RT-PCR. Data are given as relative mRNA expression compared to mRNA expression of PBS-treated control cells (control = 1.0). n = 4 (± SEM), * p<0.05, 2-tailed paired Student’s t test. (TIF) [file pone.0050518.s001.tif]

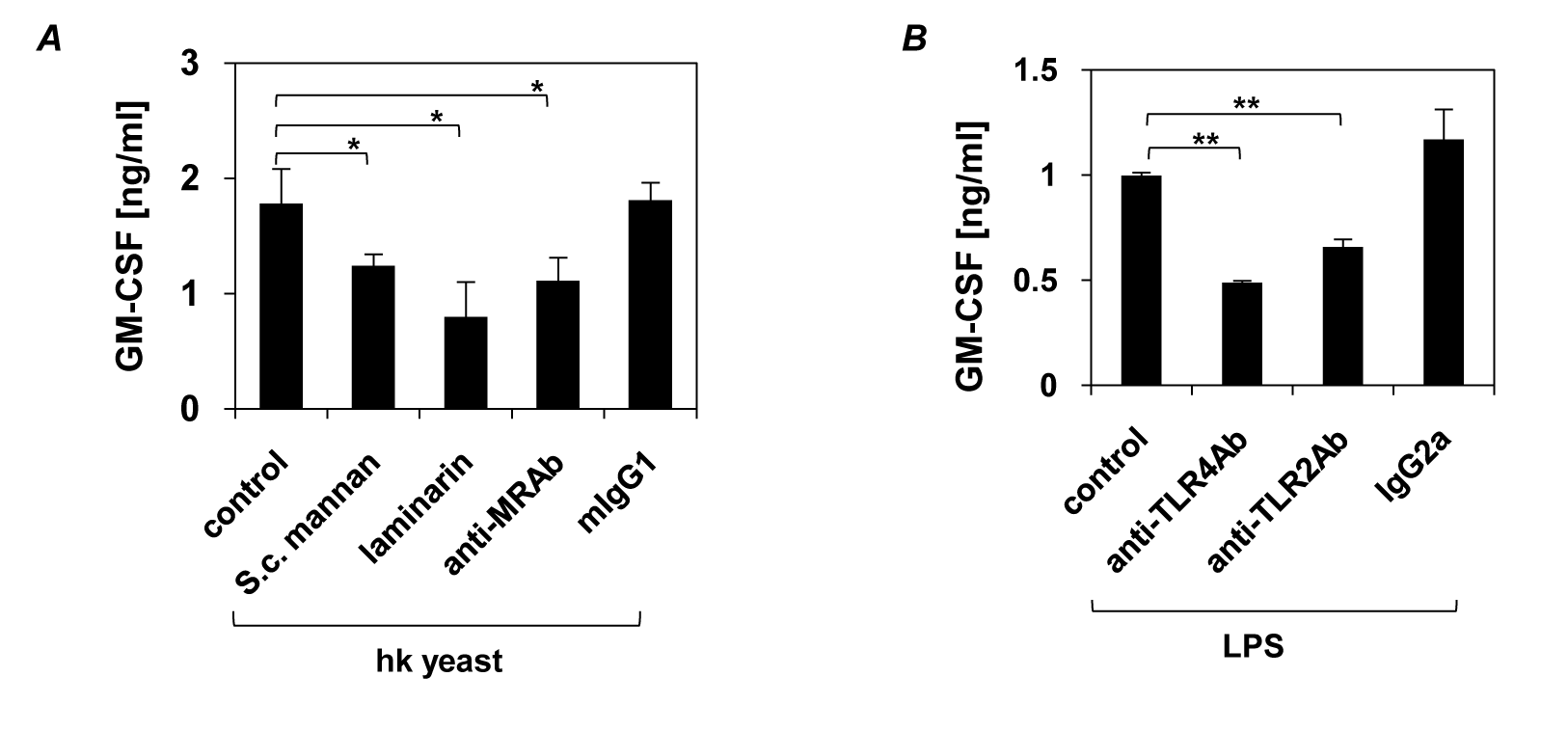

Supplement: Figure S2 — Positive blocking effect of antibodies and carbohydrates. Human PBMCs (1×10^6^) were pre-incubated with 10 µg/ml anti-TLR2, anti-TLR4, anti-MR antibodies, laminarin (100 µg/ml) or S. cerevisiae mannan (40 µg/ml) 1 h before cells were stimulated with heat-treated C. albicans (1×10^8^) for 24 h or LPS (100 ng/ml). GM-CSF was quantified by ELISA. n = 3 (± SEM), * p<0.05, **p<0.01, 2-tailed paired Student’s t test. (TIF) [file pone.0050518.s002.tif]
